# Review of Instrumented Indentation

**DOI:** 10.6028/jres.108.024

**Published:** 2003-08-01

**Authors:** Mark R. VanLandingham

**Affiliations:** National Institute of Standards and Technology, Gaithersburg, MD 20899-8610

**Keywords:** calibration methods, contact mechanics, depth-sensing indentation, elastic modulus, instrumented indentation, measurement science, nanoindentation, tip shape characterization.

## Abstract

Instrumented indentation, also known as depth-sensing indentation or nanoindentation, is increasingly being used to probe the mechanical response of materials from metals and ceramics to polymeric and biological materials. The additional levels of control, sensitivity, and data acquisition offered by instrumented indentation systems have resulted in numerous advances in materials science, particularly regarding fundamental mechanisms of mechanical behavior at micrometer and even sub-micrometer length scales. Continued improvements of instrumented indentation testing towards absolute quantification of a wide range of material properties and behavior will require advances in instrument calibration, measurement protocols, and analysis tools and techniques. In this paper, an overview of instrumented indentation is given with regard to current instrument technology and analysis methods. Research efforts at the National Institute of Standards and Technology (NIST) aimed at improving the related measurement science are discussed.

## 1. Introduction

Indentation or hardness testing has long been used for characterization and quality control of materials, but the results are not absolute and depend on the test method. In general, traditional hardness tests consist of the application of a single static force and corresponding dwell time with a specified tip shape and tip material, resulting in a hardness impression that has dimensions on the order of millimeters. The output of these hardness testers is typically a single indentation hardness value that is a measure of the relative penetration depth of the indentation tip into the sample. For example, the Rockwell hardness scales are distinguished by the amount of static force applied (e.g., 100 kg for Rockwell B compared to 150 kg for Rockwell C); the tip shape (e.g., a spherical tip with a diameter of 1.6 mm for Rockwell B compared to a conical tip that has a spherical apex with a radius of 200 µm for Rockwell C); and the tip material (e.g., steel for Rockwell B compared to diamond for Rockwell C) [1]. Harder metals are more appropriately characterized using higher forces and/or pyramidal tips, whereas lower forces and spherical tips are used on softer metals. Similarly, different durometers, which are used to characterize the mechanical resistance of polymers, have different spring constants and either a flat conical tip, a sharp conical tip, or a spherical tip, as specified in ASTM D 2240 Standard Test Method for Rubber Property—Durometer Hardness. Durometers with higher spring constants and sharp tips can be used to evaluate stiffer polymers compared to durometers with lower spring constants and/or flat or spherical tips.

Instrumented indentation, also known as depth-sensing indentation or nanoindentation, is increasingly being used to probe the mechanical response of materials from metals and ceramics to polymeric and biological materials. In contrast to traditional hardness testers, instrumented indentation systems allow the application of a specified force or displacement history, such that force, *P*, and the displacement, *h*, are controlled and/or measured simultaneously and continuously over a complete loading cycle. Additionally, the extremely small force and displacement resolutions, often as low as ≈1 µN and ≈0.2 nm, respectively, or lower for some systems, are combined with very large ranges of applied forces and displacements (tens of µN to hundreds of mN or larger in force and tens of nm to tens of µm or larger in displacement) to allow a single instrument to be used to characterize nearly all types of material systems. Recent developments include improved user control over loading histories, improved system and software robustness allowing much higher levels of test automation, and the use of dynamic oscillation for improved sensitivity and additional testing capabilities.

The additional levels of control, sensitivity, and data acquisition offered by instrumented indentation systems have resulted in numerous advances in materials science, particularly regarding fundamental mechanisms of mechanical behavior at micrometer and even sub-micrometer length scales. Instrumented indentation systems have been used to study, for example, dislocation behavior in metals [2–4], fracture behavior in ceramics [5–6], mechanical behavior of thin films [7–10] and bone [11], residual stresses [12], and time-dependent behavior in soft metals [13–15] and polymers [16–22]. Additionally, lateral probe motion is also being incorporated to explore tribological behavior of surfaces, including scratch resistance of coatings [23, 24] and wear resistance of metals [25]. The extent to which these techniques can be used to quantify macroscopic material properties is continually being explored. Indentation values of elastic modulus are routinely calculated and compared to values from macroscopic mechanical tests. Further, a number of researchers are utilizing techniques such as dimensional analysis and finite element modeling to relate material constitutive behavior to indentation force-displacement data, and even to predict stress-strain behavior based on instrumented indentation data [26, 27].

Continued improvements of instrumented indentation testing towards absolute quantification and standardization for a wide range of material properties and behavior will require advances in instrument calibration, measurement protocols, and analysis tools and techniques. In this paper, an overview of instrumented indentation is given with regard to current instrument technology and analysis methods. Research efforts at the National Institute of Standards and Technology (NIST) are then discussed, which are aimed at improving the related measurement science.

## 2. Overview of Instrumented Indentation

### 2.1 Instrumentation

**Fig. 1:**
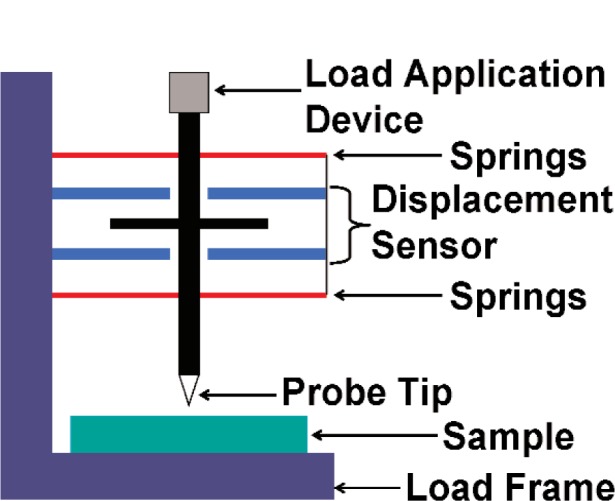
Schematic illustration of an instrumented indentation system.

Many instrumented indentation systems can be generalized in terms of the schematic illustration shown in Fig. 1 [28,29]. Commercially available systems include those developed by Nano Instruments^1^ (Oak Ridge, TN; now part of MTS Systems Corp.) [28], Hysitron, Inc. (Minneapolis, MN) [30], Micro Materials Ltd. (UK) [31], CSIRO (Australia) [32], and CSEM (now CSM) Instruments (Switzerland) [23]. Force is often applied using either electromagnetic or electrostatic actuation, and a capacitive sensor is typically used to measure displacement. In the MTS Nano Instruments, Hysitron, CSIRO, and CSM Instruments systems, the axis of the indenter is vertical (see Fig. 1), while in the Micro Materials system, it is horizontal. Also, the MTS Nano Instruments, Micro Materials, CSIRO, and CSM Instruments systems apply force and measure displacement through separate means, while the Hysitron system uses the same transducer for both force application and displacement measurement. Regardless of the operational differences, the raw force and raw displacement data for these systems are always coupled due to the leaf springs, and in fact, a plot of the raw force as a function of the raw displacement with the tip out of contact is characteristic of the spring stiffness. In the following sections, the measurement methods, analysis techniques, and calibration issues presented are applicable, in general, to current commercial instrumented indentation systems.

### 2.2 Measurement Methods

**Fig. 2:**
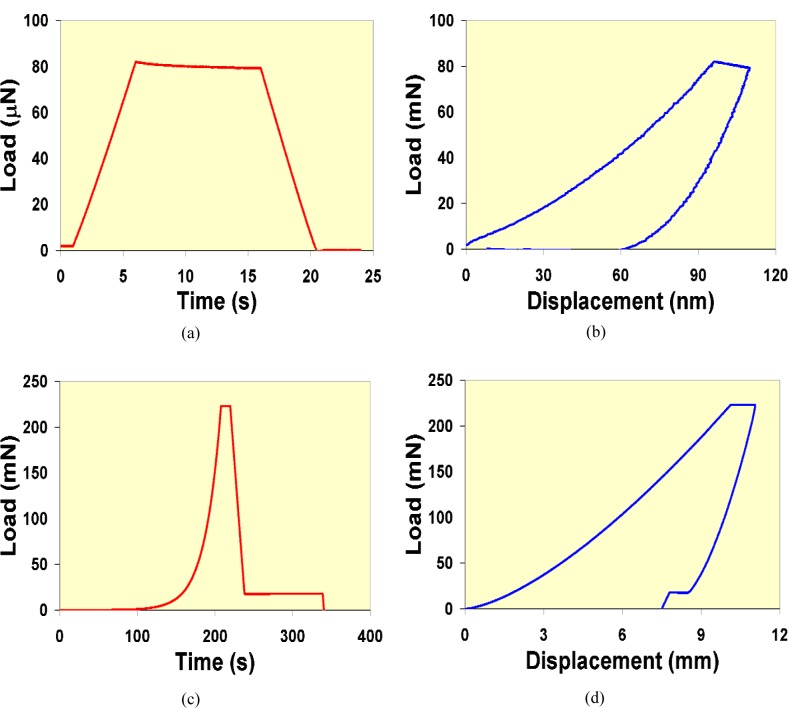
Force-time (a and c) and corresponding force-displacement (b and d) indentation data: (a and b) data taken on poly(methyl methacrylate) using a constant $\dot{P}$ loading; (c and d) data taken on polystyrene using constant $\dot{P}/P$ loading. Both data sets include a 10 s hold period between loading and unloading, and the data in (c) and (d) include an additional hold period near the end of the unloading segment.

Some of the earliest research related to the development of instrumented indentation is found in the early-to mid-1980’s [29, 31, 33, 34]. Since that time, the continuing evolution of computer technology has led to improved control over instrumented indentation testing. Although some specialized research instruments are displacement controlled (for example, the inter-facial force microscope [35]), most indentation systems are force-driven devices, such that the force history is most easily controlled with displacement control achieved using signal feedback. Typical force-time (*P-t*) and force-displacement (*P-h*) data are shown in Fig. 2. In Fig. 2a and Fig. 2b, indentation data taken using a constant loading rate, $\dot{P}$, is shown for poly (methyl methacrylate), whereas in Fig. 2c and Fig. 2d, loading data are shown for constant $\dot{P}/P$ for polystyrene. In both cases, the loading segment is followed by a hold segment, which is then followed by an unloading segment using a constant unloading rate. In Fig. 2c and Fig. 2d, a hold period near the end of the unloading segment is also shown. Assuming the mechanical behavior of the material is not a function of time, the ratio of loading rate, $\dot{P}$, to force, *P*, has been related to a proposed expression for strain rate for an indentation measurement, ${\dot{\varepsilon }}_{i}$, through the following relation [36]:

$${\dot{\varepsilon }}_{i}=\frac{\dot{h}}{h}=\frac{1}{\beta }\left(\frac{\dot{P}}{P}-\frac{\dot{H}}{H}\right)$$ (1)

Here, *H* = *P/A* is the hardness, $\dot{H}=dH/dt$ is the time rate of change during testing of the hardness, and *β* describes the shape of an idealized indentation tip, where the cross-section area, *A*, is related to the distance, *h*, from the tip apex by *A* = ch*^β^*. Note that in Eq. (1), *β* cannot be 0, so that the right side of this equation is not valid for flat punch geometry. For a tip-sample combination for which *H* is constant with depth, constant $\dot{P}/P$ testing yields a constant indentation strain rate. For many materials, *H* is a function of ${\dot{\varepsilon }}_{i}$, which can then be characterized using these types of tests. Further, because *H* = *P/A* represents a mean pressure or stress during indentation, constant $\dot{P}/P$ testing could be used, for example, to study creep behavior under “constant H” (or equivalently, “constant mean pressure” or “constant mean stress”) conditions in time-dependent materials.

A more ubiquitous test method used in force-controlled instrumented indentation employs the use of a constant loading rate, or for a displacement-controlled system, a constant displacement rate. In either case, no feedback is necessary, which lends to the simplicity of these methods. Of course, feedback could be used to produce a constant displacement rate in a force-controlled system or a constant loading rate in a displacement-controlled system. The former approach is perhaps more useful, because displacement, *h*, and displacement rate, $\dot{h}$, are linked to indentation strain and strain rate, whereas force and loading rate are more difficult to link to either stress or strain. For example, for conical or pyramidal tip geometry, a nominal indentation strain is related to the characteristic included angle or angles of the tip. For a paraboloidal tip, indentation strain is related to the ratio of the contact radius to the tip radius, and the contact radius is directly related to *h* [16, 21]. For any tip geometry, the indentation strain rate can be calculated from the ratio $\dot{h}/h$, as in Eq. (1). However, the mean stress or hardness, *H*, is the ratio of force, *P*, to contact area, *A*, which in general is related to displacement through the tip geometry. Only in the case of a flat punch, where *A* is constant with *h*, is *H* a function of force only. As shown in Eq. (1), $\dot{P}/P$ can be related to indentation strain rate, but only through additional calculations of $\dot{H}$ and *H*.

**Fig. 3:**
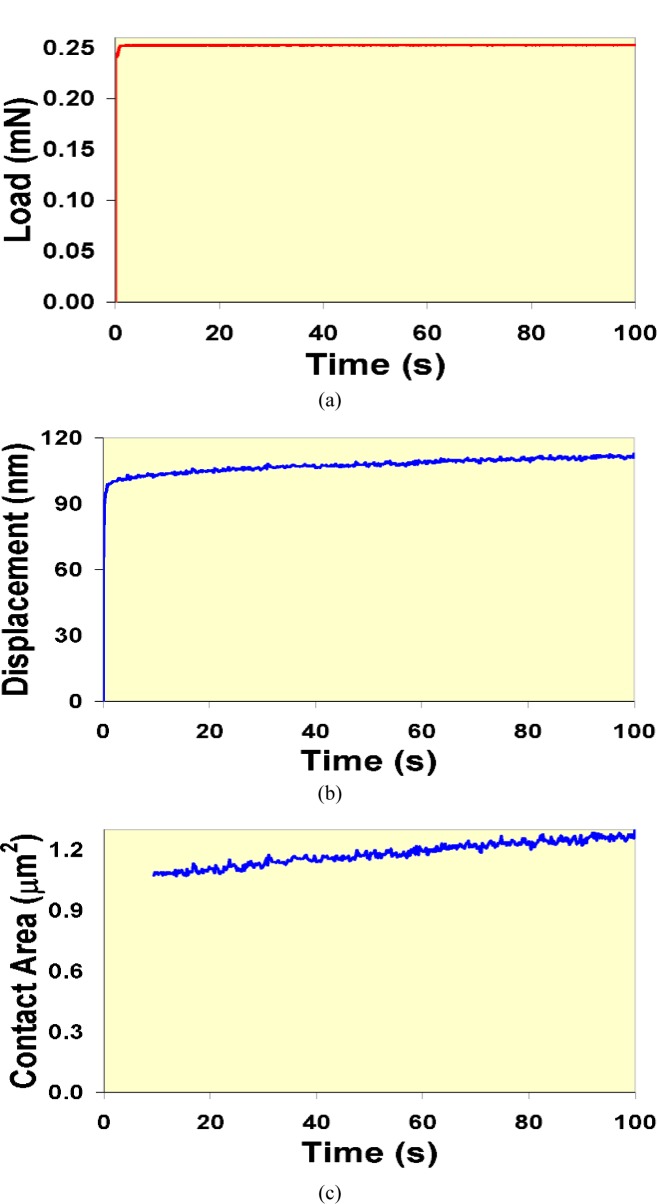
Force-time (a) and corresponding displacement-time (b) and contact area-time data (c) for an indentation creep test using a force-controlled system.

**Fig. 4:**
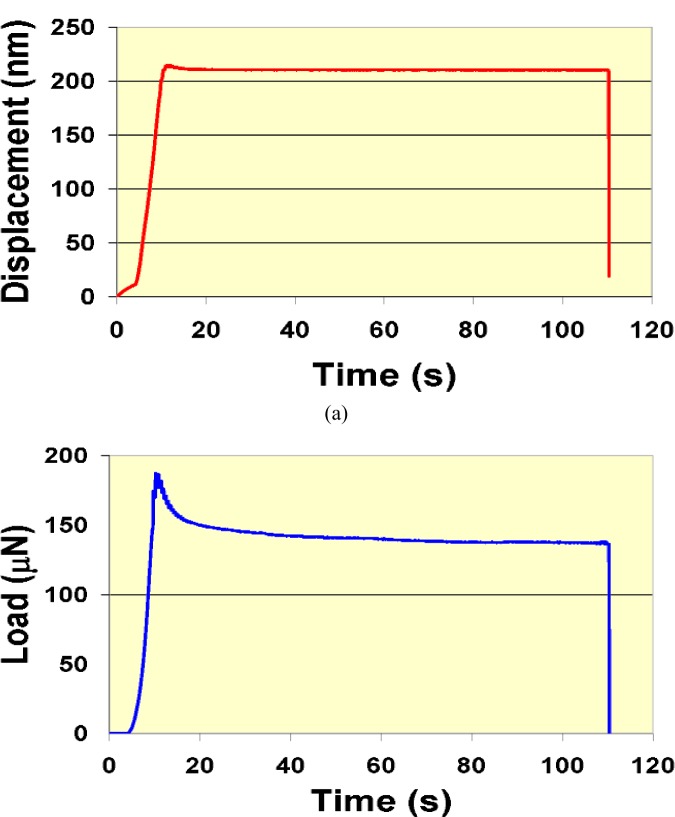
Displacement-time (a) and corresponding force-time (b) for an indentation stress-relaxation test using a force-controlled system and feedback to control displacement.

Other quasi-static test methods that have been used to characterize time-dependent response are indentation creep tests [13–15, 17, 21] and indentation stress relaxation tests [21]. As in a traditional tensile creep test, force is held constant in an indentation creep test and displacement or strain is monitored [1]. However, for the case of tensile creep, the change in cross-section area as the sample elongates is typically small during most of the test, such that constant force is essentially equivalent to constant stress. In the indentation creep test, the gradual displacement of the tip into the sample causes a substantial change in the contact area, as shown in Fig. 3, such that both the stress and the strain fields evolve during the test, complicating analysis and comparison to bulk measurements. For the indentation stress relaxation test, displacement is held constant (through feedback in a force-controlled system) and the gradual decrease in force is recorded. Because displacement is closely linked to strain in an indentation experiment, constant indentation displacement gives constant indentation strain. Thus, data from this type of measurement method are more easily analyzed and compared to traditional bulk stress relaxation data. However, because very fast initial force and displace ment rates are normally used in creep and stress relaxation tests, respectively, the use of feedback combined with instrument limitations typically causes issues regarding how fast the force or displacement can be applied and then held constant without substantial overshooting or undershooting, which can affect the quality particularly of the short-time data. An example of indentation stress relaxation data taken on a force-controlled system is shown in Fig. 4 to illustrate this problem.

**Fig. 5:**
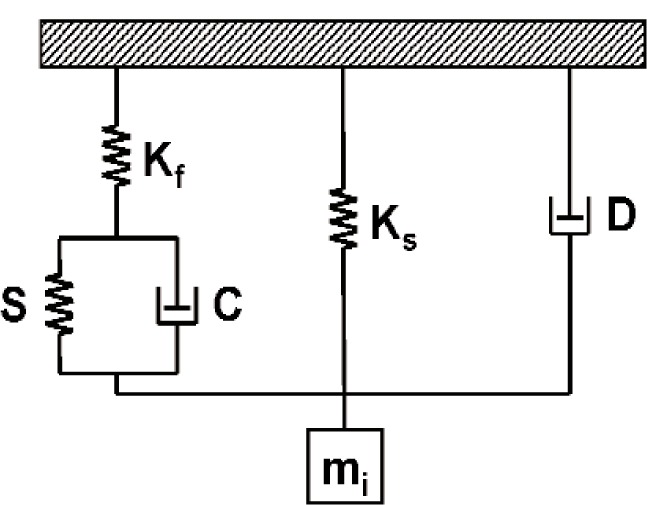
Schematic illustration of a dynamic model for an instrumented indentation system. *K*_f_ represents the load-frame stiffness, *K*_s_ represents the stiffness of the springs, *D* and *m*_i_ represent the damping characteristics and mass, respectively, of the instrument, and *S* and *C* represent the storage and loss components, respectively, of the mechanical impedance related to the tip-sample contact.

Recently, test methods have been used that combine dynamic oscillation with the quasi-static testing capabilities of instrumented indentation systems. A typical dynamic model of the indentation system represented in Fig. 1 is shown schematically in Fig. 5. When dynamic oscillation is applied, it is most often superposed over a quasi-static force history using a small force or displacement amplitude and a frequency in the range of 1 Hz to 300 Hz [19, 37]. As will be discussed further in Sec. 2.3, the equations developed for the dynamic model are used to determine the contact stiffness throughout the force history, which in turn allows the contact area and sample modulus to be estimated throughout the force history. This technique is thus particularly useful for characterizing modulus as a function of depth, estimating changes in contact area during an indentation creep test (see Fig. 3c), and exploring the storage and loss responses of materials, for example. Additionally, the statistical sampling of indentation testing is dramatically improved over getting one set of values for a given test from analysis of the unloading curve slope (see Sec. 2.3). Also, certain parameters related to the system dynamics will change dramatically at the onset of tip-sample contact, significantly improving the ability to determine this point in the indentation data.

### 2.3 Analysis Techniques

The analysis of force-displacement curves produced by instrumented indentation systems is often based on work by Doerner and Nix [29] and Oliver and Pharr [28]. Their analyses were in turn based upon relationships developed by Sneddon [38] for the penetration of a flat elastic half space by different probes with particular axisymmetric shapes (e.g., a flat-ended cylindrical punch, a paraboloid of revolution, and a cone). These elasticity-based analyses are normally applied to the unloading data of an indentation measurement, assuming the unloading behavior of the material is characterized by elastic recovery only. In general, the relationships between penetration depth, *h*, and force, *P*, during unloading can be represented in the form

$$P=\alpha {\left(h-h_{f}\right)}^{m}$$ (2)

The parameter *α* contains geometric constants, the sample elastic modulus, *E*, the sample Poisson’s ratio, *ν*, the indenter elastic modulus, *E*_i_, and the indenter Poisson’s ratio, *ν*_i_; the parameter *h*_f_ is the final unloading depth; and *m* is a power law exponent that is related to the geometry of the indenter (see Table 1). A nonlinear power law fit to the unloading data, where *α*, *h*_f_, and *m* are fitting parameters, often yields a good estimate of the data, as does a smooth spline fit [20]. However, previous research at NIST has shown that the practice of arbitrarily fitting portions of the unloading data can introduce bias into the calculations, and that data should be removed for curve fitting purposes only when the corresponding residual errors do not conform to the assumptions underlying the curve fitting methods [20]. Once an appropriate fit is obtained, a derivative, d*P*/d*h*, applied at the maximum loading point (*h*_max_, *P*_max_) should yield information about the state of contact at that point. This derivative is termed the contact stiffness, *S*, and is given analytically by

$$S=2aE_{r}=\frac{2}{\sqrt{\pi }}E_{r}\sqrt{A.}$$ (3)

In this equation, the cross section of the indenter is assumed to be circular in relating the contact radius, *a*, to the projected area of tip-sample contact, *A*. A small correction is sometimes applied for non-circular cross sections [26] and other corrections have been suggested [39]. The reduced modulus, *E*_r_, accounts for elastic deformation of both the indenter and the sample and is given by

$$\frac{1}{E_{r}}=\frac{\left(1-\nu^{2}\right)}{E}+\frac{\left(1-\nu_{i}^{2}\right)}{E_{i}}.$$ (4)

**Fig. 6:**
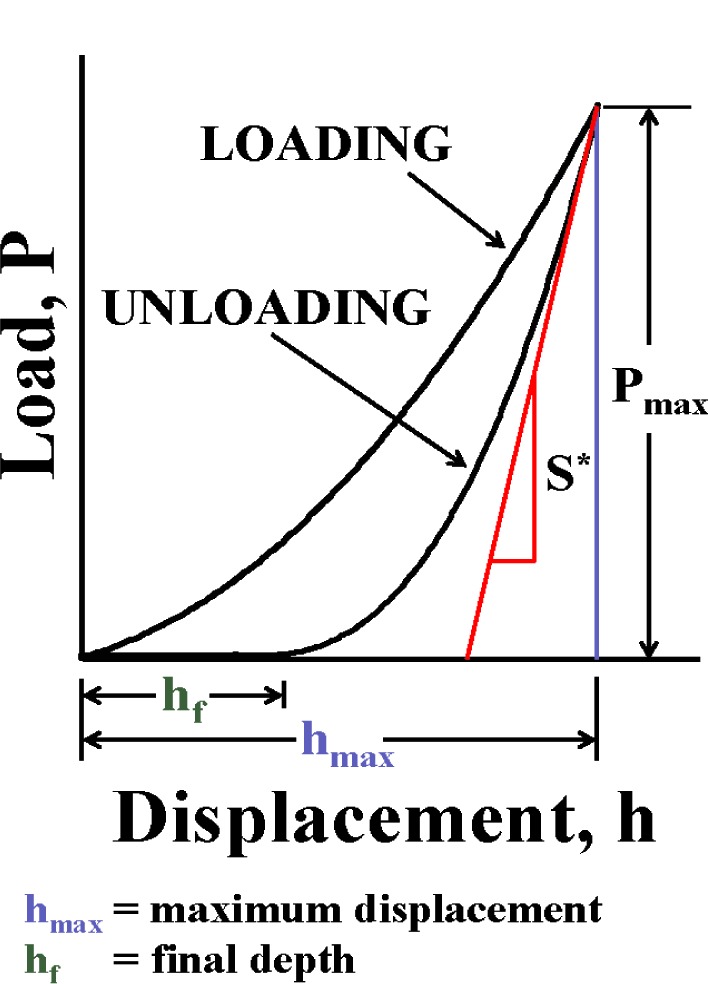
An indentation force-displacement curve in which several important parameters used in the Oliver and Pharr analysis are illustrated.

In Fig. 6, an indentation force-displacement curve is illustrated along with several important parameters used in the Oliver and Pharr analysis. The measured stiffness, S^*^, is the slope of the tangent line to the unloading curve at the maximum loading point (*P*_max_) and is given by

$$S^{*}={\left(\frac{dP}{dH}\right)}_{P_{\max}}=am{\left(h_{\max}-h_{f}\right)}^{m-1}.$$ (5)

**Fig. 7:**
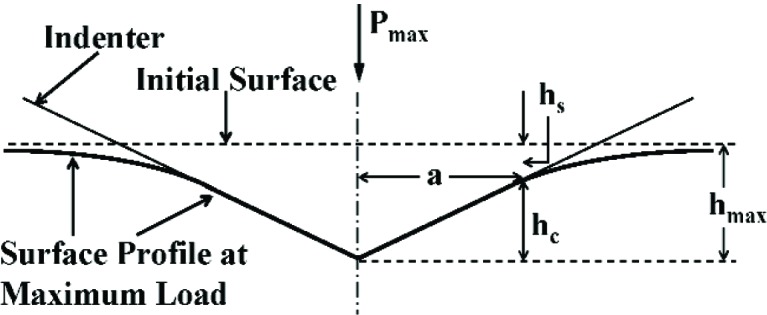
Illustration of the indentation geometry at maximum force for an ideal conical indenter.

The parenthetic subscript denotes that the derivative is evaluated at the maximum loading point. Physically, the elastic recovery of the material is instantaneous upon unloading, so the true elastic response of the material can only be evaluated at *t* = 0^+^, where unloading initiates at *t* = 0. When the displacement, *h*, is the total measured displacement of the system, S^*^ is the total system stiffness. After successful calibration of the load-frame compliance (see Sec. 2.4), the displacement of the load frame is removed so that *h* represents only the displacement of the tip into the sample, and S^*^= *S*. The contact depth, *h*_c_, is related to the deformation behavior of the material and the shape of the indenter, as illustrated in Fig. 7, and is given by *h*_c_ = *h*_max_− *h*_s_, where *h*_s_ is defined as the elastic displacement, sometimes referred to as sink-in, of the surface at the contact perimeter. For each of three specific tip shapes (flat-ended punch, paraboloid of revolution, and cone), *h*_s_ = *ε P*_max_/*S* where *ε* is a function of the particular tip geometry, as summarized in Table 1. Thus, *h*_c_ is given by

$$h_{c}=h-\frac{\varepsilon P}{S}.$$ (6)

For the purposes of the Oliver-Pharr analysis, *h* = *h*_max_ and *P* = *P*_max_ in Eq. (6). Also, *h*_c_ < *h*_max_ such that this equation is not valid when pile-up occurs, i.e., when material is forced up along the sides of the indentation tip. As indicated in Table 1, the choice of *ε* should be related to the value of *m* determined from the curve fitting [40]. However, a value of 0.75, corresponding to *m* = 1.5, is used almost exclusively for spherical, conical, and pyramidal indenters, regardless of the value of *m*. Once *h*_c_ has been determined, the tip shape function, *A*(*h*_c_) (see Sec. 2.4) is used to calculate the contact area, such that the sample modulus, *E*, can be determined from Eq. (3) and Eq. (4), given a reasonable estimate of *ν*.

Despite the use of feedback in some cases, the dynamic model of Fig. 5 is normally treated as a simple damped harmonic oscillator under conditions of an applied harmonic force, *P* = *P*_0_ · exp (*iω t*), with a resulting harmonic displacement, *h* = *h*_0_ · exp (*iω t − ϕ*), where *ω* is the applied oscillation frequency and *ϕ* is the phase angle by which the displacement lags the force. The equations of motion developed for this model (with sample contact) can be solved to yield an equation for the contact stiffness, *S*, that is a function of previously calibrated instrument parameters (i.e., *K*_f_, *K*_s_, and *m*_i_), *ω, ϕ*, the harmonic force amplitude, *P*_0_, and the harmonic displacement amplitude, *h*_0_:

$$S={\left[\frac{1}{\frac{P_{0}}{h_{0}}\cos\phi -\left(K_{s}-m\omega^{2}\right)}-\frac{1}{K_{f}}\right]}^{-1}.$$ (7)

Also, the damping factor, *C*, attributed to the tip-sample contact is given by:

$$C\omega =\frac{P_{0}}{h_{0}}\sin\phi -D\omega .$$ (8)

Superposing oscillation during quasi-static loading thus allows *S* to be estimated as a function of depth throughout the loading segment. Equation (6) can then be used to monitor *h*_c_ continuously, knowledge of the tip shape yields an estimate of *A* continuously, and thus *E* can be measured continuously using Eq. (3) and Eq. (4). Because these relationships can be built into the software, the use of dynamic oscillation superposed over the loading history simplifies the analysis procedures greatly. Additionally, the amount of data associated with one such experiment is equivalent to many experiments in which only quasi-static loading is used, allowing more robust statistical analysis to be performed.

### 2.4 Calibration Issues

Very little discussion of calibration issues related to instrumented indentation systems can be found in the open literature. As with most measurement systems, calibration is essential for limiting uncertainties and achieving reproducible and repeatable measurements. The most fundamental measures made by these systems are of force and displacement. Many instruments are capable of operating at forces less than 1 mN, and in some literature, force resolutions of 1 µN or less are claimed. Currently, however, force is typically calibrated by hanging standard weights on the force measurement device, and deadweight force standards are only available for calibrating forces down to approximately 10 µN. Current NIST research aimed at improving available force standards at the micro-Newton and nano-Newton levels is discussed in Sec. 3.1.

Calibration of displacement can be done using several methods, such as by using separately calibrated transducers or by using interferometric methods. Again, however, many instruments are capable of operating with displacements and displacement resolutions significantly smaller than the resolution of the calibration methods. Further, force and displacement measurements are typically coupled in instrumented indentation systems through the support springs. Thus for force-driven systems, for example, calibrating displacement is equivalent to calibrating the spring stiffness or spring constant, which links the raw displacement of the system to the raw force. Assuming the spring response is linear, displacements can then be determined with uncertainties related to uncertainties in the force calibration and the spring constant.

The calibration of load-frame compliance, *C*_lf_, is difficult and can have a significant amount of uncertainty, and even qualitative assessment of indentation behavior depends critically on the accuracy of this calibration step, especially from laboratory to laboratory and instrument to instrument. Prior to calibration (i.e., *C*_lf_ is unknown), the measured displacement, *h*_total_, is a combination of displacement of the load frame, *h*_lf_, and displacement of the sample, *h*_sp_. Treating the system as two springs (the load frame and the sample) in series under a given force, *P*,

$$h_{\mathrm{total}}=h_{lf}+h_{\mathrm{sp}}.$$ (9)

Dividing both sides by *P*,

$$C_{\mathrm{total}}=C_{\mathrm{lf}}+C_{\mathrm{sp}}=C_{\mathrm{lf}}+\frac{\sqrt{\pi }}{2E_{r}}\frac{1}{\sqrt{A}}.$$ (10)

The total compliance is related to total stiffness, S^*^, by *C*_total_ = 1/S^*^ and the sample compliance is related to the contact stiffness, *S*, by *C*_sp_ = *1*/*S*. A number of possible methods exist for determining *C*_lf_ using reference samples that are homogeneous and isotropic and for which both *E* and *ν* are known. Typically, a series of indentation measurements are made on a single reference sample or multiple reference samples. Oliver and Pharr [28] suggested using an iterative technique to calibrate both the load-frame compliance and the tip shape with one set of data from a single reference sample, as both *C*_lf_ and *A* are, in general, unknowns in Eq. (10). While this method has the advantage of not requiring an independent measurement of the area of each indent, its use has been limited, perhaps because it is mathematically and time intensive.

For the load-frame compliance calibration, the choice of reference sample(s) should be made with an objective of maximizing contact stiffness (minimizing *C*_sp_) so that *C*_total_ is dominated by *C*_lf_. Thus, relatively large indentation forces and depths are normally applied to a reference material that either has a high modulus or exhibits significant plastic deformation, i.e., a sample that has a high ratio of *E*/*H*. Use of a highly plastic material such as aluminum, for which the projected area should be similar to the contact area, *A*, at maximum force, can be combined with high resolution imaging techniques, such as electron microscopy or atomic force microscopy (AFM) to determine *C*_lf_ from a plot of *C*_total_ as a function of $1/\sqrt{A}$. Alternatively, an additional assumption that *H* is constant with depth can allow *C*_lf_ to be determined from a plot of *C*_total_ as a function of $1/\sqrt{P_{\max}}$. In this case, aluminum is often replaced by fused silica, because oxide formation on aluminum can create variations in both *E* and *H* with penetration depth. For all of these methods, the calculation of *C*_lf_ based on Eq. (10) requires a large extrapolation of the experimental data. Futher, the *x*-variable has significant uncertainty, which is a violation of the assumptions of least squares regression, leading to the creation of bias in the least squares estimate of *C*_lf_ such that the estimated value is lower than the actual value. These issues, along with other sources of uncertainty, can result in a large amount of uncertainty associated with the load-frame compliance, which will then affect all subsequent calculations [20, 41]. A review of these uncertainties along with a newly proposed method of load-frame compliance calibration that is independent of reference materials can be found in Ref. [42].

For the tip shape calibration, the series of indents applied to a reference material typically covers a larger range of maximum force and maximum penetration depth compared to the load-frame compliance calibration procedure. The objective of tip shape calibration is to estimate the cross-section area of the indenter tip as a function of distance from the apex. In Fig. 7, the indentation geometry for a conical indenter is illustrated in two dimensions. At a given force, *P*, the contact area, *A*, which is related to the contact radius, *a*, is the cross-section area of the indenter tip at a distance, *h*_c_ (the contact depth), from the tip apex. From measurements of *h*_max_, *P*_max_, and *S*, Eq. (3) and Eq. (6) can be used to calculate *A* and *h*_c_, respectively, for each indentation. A tip shape function, *A*(*h*_c_), is determined, given a sufficient number of measurements over a range of *h*_c_ values, by fitting the *A* vs *h*_c_ data, typically using a multi-term polynomial fit of the form:

$$A\left(h_{c}\right)=B_{0}h_{c}^{2}+B_{1}h_{c}+B_{2}h_{c}^{1/2}+\ldots .$$ (11)

In this equation, *B*_0_, *B*_1_,…, *B*_n_ are constant coefficients determined by the curve fit. For example, for a Berkovich indentation tip, Oliver and Pharr [28] suggested using up to nine terms (*n* = 8) with *B*_0_ = 24.5, where the area function of a perfect Berkovich tip is *A*(*h_c_*) = 24.5 *h_c_*^2^. The additional terms attempt to account for deviations from ideal geometry, such as blunting of the tip.

The use of dynamic oscillation can significantly enhance the calibrations of load-frame compliance and tip shape, particularly with regard to improving the statistical sampling and reducing the amount of time required. For example, multiple deep indentations on a reference sample using oscillation superposed over the loading segment of each test yields multiple sets of modulus values as a function of penetration depth that can then be averaged and used either to check load-frame compliance or tip shape calibration. In fact, both can at least be checked with the same data if the reference sample (e.g., fused silica glass) has both a modulus and a hardness that is independent of depth. Thus, the *E* vs *h* data can be used to check tip shape calibration, and for a proper load-frame compliance calibration, the ratio of force to contact stiffness squared (P/*S*^2^), which is proportional to *H*/*E*_r_, should be independent of depth regardless of the tip shape function. Additionally, the improved capabilities for detecting the surface using dynamic oscillation reduce the uncertainties in the calibrations related to the choice of the initial point of contact.

Dynamic calibrations of the system are typically made with respect to the dynamic model shown in Fig. 5 [37]. System calibrations are then performed by measuring the dynamic response with no sample involved. The load-frame stiffness, *K*_f_, is normally assumed to be infinite (i.e., load-frame compliance is zero). By monitoring amplitude and phase shift, the equations derived for the model can be used to determine the resonance frequency of the system, the system damping coefficient, *D*, the mass, *m*_i_, and the spring constant, *K*_s_. As discussed previously, the spring constant, which is typically independent of frequency over a wide frequency range, can be determined from raw force as a function of raw displacement, and the system mass can be determined from the displacement at zero force. Also, *D* is often assumed to be independent of frequency, which is not necessarily true [43].

## 3. Research Efforts at NIST

### 3.1 Improving Force Calibration

A desire for accurate, traceable, small force measurement is emerging within the International Organization for Standardization (ISO) task groups and ASTM International technical committees that work on instrumented indentation standards [44]. However, no methods for establishing force measurement traceability at these levels are currently available. A research effort has recently been developed at NIST (Microforce Realization Competence) with the purpose of creating a facility and instruments capable of providing a viable primary force standard below 10^−5^ N, and with the goal of realizing force in this range at a relative uncertainty of parts in 10^4^. This new project complements a body of existing work at NIST to develop standards and methods for the instrumented indentation community that together provide a metrological basis for manufacturers seeking traceable characterization of, for example, thin film mechanical properties.

The most common approach to force realization is a calibrated mass in a known gravitational field or deadweight force, which is universally accepted as the primary standard of force. The smallest calibrated mass available from NIST is 1 mg (approximately a 10 µN deadweight force) having a relative uncertainty of about 10^−4^. In principle, smaller masses could be calibrated, but they would be difficult to handle. Also, the relative uncertainty tends to increase in verse proportion to the decrease in mass [45], potentially resulting in uncertainties that are of similar magnitude to deadweight forces in the range of nano-Newtons.

Alternatively, forces in this range can be realized using the electrical units defined in the International System of Units (SI) and linked to the Josephson and quantized Hall effects in combination with the SI unit of length. This realization can be done using electromagnetic forces (e.g., the NIST Watt Balance Experiment [46]) or using electrostatic forces [47]. In this research, the latter was chosen because the required metrology is somewhat simpler to execute, and the forces generated, although generally less than those feasible electromagnetically, are appropriate for the force range of interest. Also, electrostatic force generation is common in micro-electromechanical systems (MEMS), and the ability to calibrate such forces from electrical and length measurements could prove beneficial.

The mechanical work required to change either the overlap or the separation of two electrodes in a one-dimensional capacitor while maintaining constant voltage is

$$dW=F\cdot dz=\frac{1}{2}V^{2}dC.$$ (12)

In this equation, d*W* is the change in energy (mechanical work), *F* is the force, d*z* is the change in the overlap or separation of the electrodes, *V* is the electric potential across the capacitor, and d*C* is the change in capacitance. Thus, force can be realized from electrical units by measuring *V* and the capacitance gradient, d*C*/d*z* or:

$$F=\frac{1}{2}V^{2}\frac{dC}{dz}$$ (13)

**Fig. 8:**
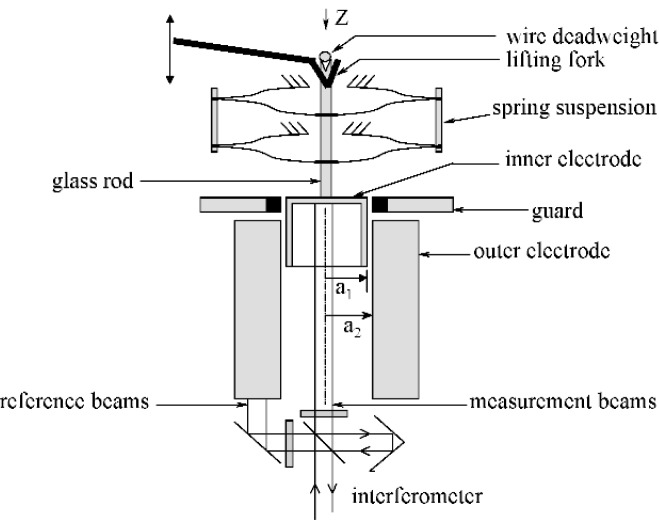
The prototype electrostatic force balance. Inner cylindrical electrode of 15 mm dia. is suspended from a compound parallelogram leaf spring made of 50 µm thick CuBe producing a single axis spring of stiffness 13.4 N/m. Deflections are measured using a double-pass Michelson interferometer and nulled using a feedback servo to apply voltage to the outer cylinder. Electrode gap is nominally 0.5 mm and overlap is nominally 5 mm.

This idealized, one-dimensional approximation does not account for multi-dimensionality, external fields or stray electrical charges likely to be present in the actual physical system. The goal then is to develop a system that reproduces this idealization as closely as possible by using effective constraints on the geometry, shielding, and suspension of the resulting electrodes. Additionally, validation of this electrostatic force realization through comparison to deadweight forces will be advantageous, at least in the higher force range where the uncertainty that can be achieved mechanically is still competitive. In consideration of these factors, a force generator was designed to operate along the vertical axis as part of an electromechanical null balance shown in Fig. 8. Recent results with this instrument [48, 49], referred to as the NIST Electrostatic Force Balance or EFB, demonstrated a relative standard uncertainty of about 10^−4^ in the comparison of gravitational and electrostatic forces ranging between 10 µN and 100 µN. This result indicates that the electrostatic force can be constrained and measured in a fashion traceable to the SI and with accuracy sufficient to warrant consideration as a primary standard of force in this regime.

### 3.2 Improving Tip Shape Calibration

A number of recent efforts have been made to improve tip shape calibration for instrumented indentation [41, 50–55]. These efforts have included material-independent methods of tip shape calibration using AFM [41, 50–52] and alternative procedures using indentation of reference materials [53–55]. Recent research at NIST has focused on methods in which the indenter tip is scanned with an AFM probe to yield direct information regarding the three-dimensional tip shape [53, 56]. Because an AFM image is a combination of the AFM probe geometry and the geometry of the sample surface, AFM imaging of indentation tips was combined with AFM imaging of a tip characterizer surface, which allows for an estimation of the three-dimensional shape of the AFM probe using the method of blind reconstruction [57, 58]. This estimation of the AFM probe geometry can then be eroded from the image of the indentation tip to remove dilation and other artifacts created by the AFM probe, as described in detail in Ref. [50].

**Fig. 9:**
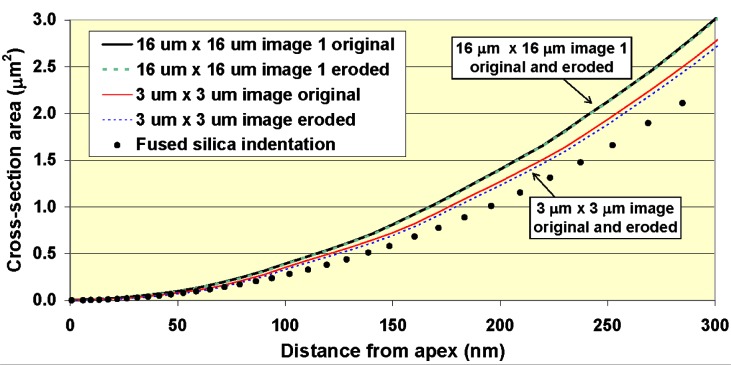
Plot of the estimated cross-section area of a Berkovich indentation tip as a function of the distance from the apex. Data are shown for two 3 µm × 3 µm images, one 16 µm × 16 µm image, each of the three AFM images after eroding away the estimated AFM probe geometry, and indentation of fused silica. Only the first 300 nm of data are shown to emphasize differences.

In principle, because the AFM probe acts to produce image geometry that is dilated from the true surface geometry, the area function (cross-section area as a function of distance from the apex) of the indentation tip determined from an AFM image is an outer bound on the true area function. Also, because the AFM probe geometry estimated using blind reconstruction is an outer bound on the true probe geometry, eroding this estimated geometry from an AFM image of the indentation tip produces a lower bound on the tip area function. In reality, however, other artifacts or uncertainties associated with the AFM image of the indentation tip will affect the results, particularly for open-loop AFM systems. This effect is shown in Fig. 9 in which area functions are compared for consecutive images of the same size and of different sizes. For close-loop AFM systems, particularly those with calibrated vertical motion such as the NIST Calibrated-AFM [59], the images produced of the tip shapes can have much less uncertainty.

Comparisons of tip shape data generated from AFM imaging to that determined from indentation of fused silica (for example, see Fig. 9) revealed a potential problem with determining tip shape area functions using indentation of reference samples. Differences between AFM-generated data and indentation data were most significant at small depths and increased as a function of the indenter tip radius. Values of cross-section area from indentation data were always less than that from AFM data. This result appears to at least partially explain results in which indentation modulus $\left(E\propto S/\sqrt{A}\right)$ is significantly higher than modulus values measured with other techniques, particularly for polymers where *S* is relatively small for a given contact depth such that errors in *A* have a larger effect and low values of *A* will result in artificially high modulus values. Also, this results could also explain the many reports of indentation hardness (*H* = *P/A*) values being larger at small depths compared to large depths, often referred to as the indentation size effect. Thus, the uncertainties associated with tip shape calibration using indentation of reference samples are expected to be significantly larger than those associated with AFM-generated tip shape information in many cases. In fact, this independent assessment of the tip shape via AFM can then be used to help calibrate load-frame compliance using a known tip area function.

### 3.3 Applications of Instrumented Indentation to Polymeric Materials

Instrumented indentation is increasingly being used to probe the mechanical response of polymeric and biological materials. These types of materials behave in a viscoelastic fashion, i.e., they display mechanical properties intermediate between those of an elastic solid and a viscous fluid, as shown in Fig. 2 and Fig. 3 by the changes in displacement under constant force conditions and in Fig. 4 by the changes in force under constant displacement conditions. The mechanical behavior is thus dependent on the test conditions, including amount of strain, strain rate, and temperature. Often in instrumented indentation, however, properties are measured using loading histories developed for elastic and elasto-plastic materials, the properties of which are not particularly time dependent, and the analysis of the indentation response is typically based on elasticity theory. In studies in which attempts have been made to characterize viscoelastic behavior, limiting and sometimes invalid assumptions have been made, and linear viscoelasticity has been applied despite the intense strains local to the indenter tip that would appear to violate the linear viscoelasticity premise of infinitesimal strains [60].

**Fig. 10:**
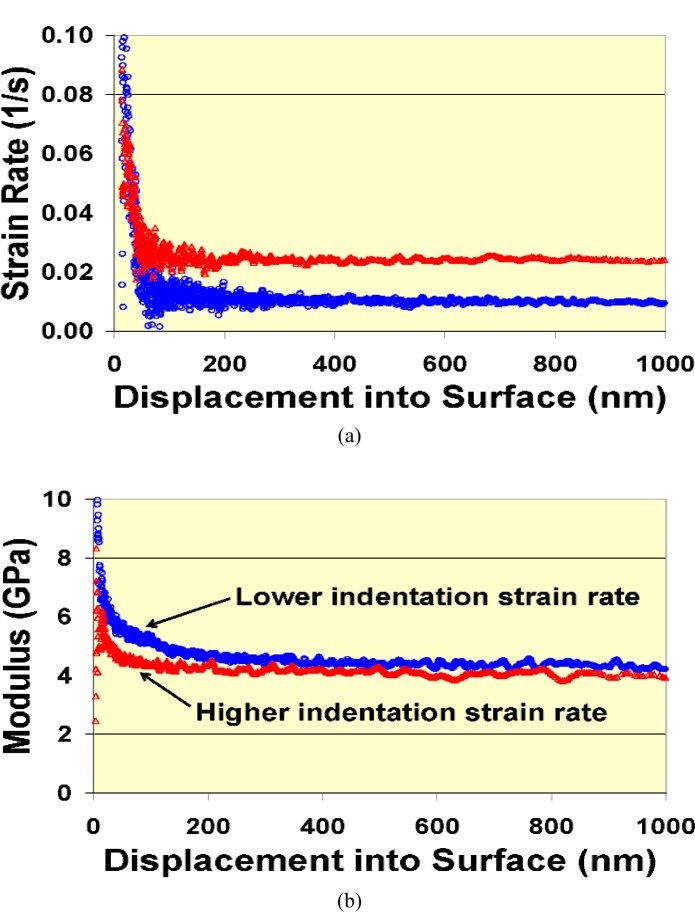
Plots of indentation strain rate, ${\dot{\varepsilon }}_{i}$, estimated by the ratio $\dot{P}/P$ (a) and indentation modulus (b) as a function of depth, *h*, for a constant $\dot{P}/P$ test using a Berkovich indentation tip to penetrate a polystyrene sample. Date for one test each at two different rates are shown. Differences in the modulus data are estimated to be within the measurement uncertainty.

Another issue that can add significant uncertainty to indentation measurements of polymers is the ability to detect the surface. Polymers and other organic materials are typically much more compliant compared to the metallic and ceramic types of materials to which instrumented indentation mainly has been applied, with modulus values ranging from a few GPa for common glassy polymers to a few MPa or lower for rubbery polymers and many biological materials. As mentioned previously, the use of small dynamic oscillations has improved sensitivity to surface contact. However, as the compliance of the material increases, depending on the particular indentation system, the changes used to define surface contact become of similar magnitude to the noise level. In some cases, manual selection of the contact point in the raw test data after the test is completed can be used to correct any automated selection by the system or to check the sensitivity of the calculations to this choice. However, in other cases, the system selection of the contact point affects the start of the test and thus the start of any feedback that might be used to control the test flow. In such cases, the approach velocity of the probe can be an important factor, as the initial part of the indentation data prior to feedback control will be based on this approach velocity. For example, the indentation strain rate, ${\dot{\varepsilon }}_{i}$, as estimated by the ratio $\dot{P}/P$, and modulus are plotted in Fig. 10 as functions of penetration depth for a polystyrene material. The feedback used to keep $\dot{P}/P$ constant does not take effect until the probe is over 100 nm into the material. Although other factors contribute to significant uncertainty at small depths, the large changes in indentation strain rate could also have affected the resulting modulus values.

In recent research at NIST, analyses by Ting [61] that are based on contact between a rigid indenter and a linear viscoelastic material were revisited and used to determine under what conditions, if any, instrumented indentation can be used to measure linear viscoelastic behavior for a number of different polymers [58]. For example, the creep compliance, *J*(*t*), of a linear viscoelastic material subject to a constant indentation or creep force, *P*_0_, using a conical tip of semi-apical angle, *θ*, is proportional to the change in contact area with time, *A*(*t*) [61]:

$$J\left(t\right)\propto \frac{A\left(t\right)}{P_{0}\;\tan\theta }.$$ (14)

Note that in this equation, (1/tan *θ*) is related to a nominal indentation strain and *P/A* is related to a nominal indentation stress, such that *J*(*t*) is proportional to an indentation strain over an indentation stress. Similar equations can be determined for other tip geometries, and equations can be derived relating stress relaxation modulus to the change in force with time during a constant displacement indentation test for various tip geometries.

**Fig. 11:**
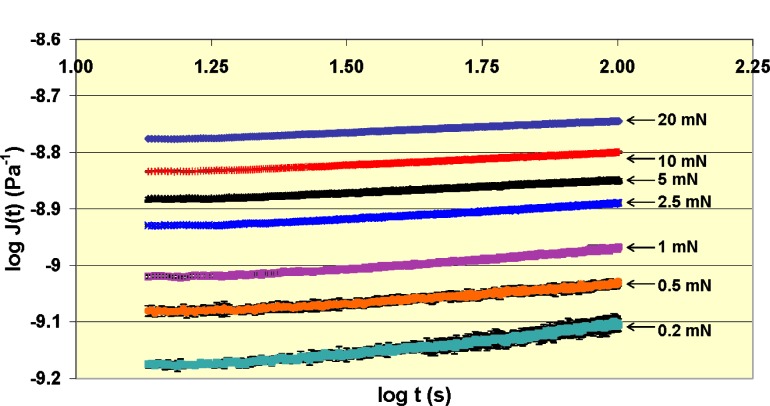
Log-log plot of creep compliance, *J*(*t*), as a function of time, *t*, for an indentation creep experiment on epoxy using a rounded conical tip (manufacturer-determined tip radius of 10 µm). Error bars shown represent an estimated standard deviation (*k* = 1).

In these studies, constant force indentation creep tests and constant penetration depth stress relaxation tests were used, and the results were compared to traditional solid rheological measurements [56]. An example of indentation creep compliance measurements for an epoxy material using a rounded conical tip (manufacturer-determined tip radius of 10 µm) is shown in Fig. 11 [proportionality constant of 1.0 assumed in Eq. (14)]. The dependence of creep compliance on the creep force, as shown by the displacement between the curves, is an indication of nonlinear behavior, and in fact, the indentation creep behavior of a number of polymeric materials was dominated by nonlinear viscoelastic behavior. Additionally, probe tip size and shape were altered to produce different nominal indentation strains, *ε_i_*, and the measured responses appeared to be correlated with *ε_i_*. Analyses and protocols are currently being explored for relating instrumented indentation data to viscoelasticity and ultimately to stress-strain behavior of polymers using various indentation tips.

In addition to the quasi-static studies, measurements of viscoelastic behavior using dynamic indentation techniques are also being explored. From the dynamic model of the indentation system (see Fig. 5), equations have been derived for determining the storage modulus, *E′*, and loss modulus, *E″*, of a viscoelastic material [18, 19, 37]:

$$E^{\prime }=\frac{\sqrt{\pi }}{2\sqrt{A}}S,$$ (15)

$$E^{\prime \prime }=\frac{\sqrt{\pi }}{2\sqrt{A}}C\omega .$$ (16)

Thus, *E*′ is assumed to be directly related to the storage portion, *S*, of the mechanical impedance of the tip-sample contact in the dynamic model (see Fig. 5), and *E*″ is assumed to be directly related to the loss portion, *Cω*, of the mechanical impedance of the tip-sample contact. However, this assumption was found to have significant limitations with regard to lossy polymers [43]. At NIST, new analysis methods are currently being developed for analyzing the dynamic mechanical response to indentation of viscoelastic materials, in particular to determine under what conditions Eq. (15) and Eq. (16) hold. A number of different polymers are being studied, the results of which are being compared to traditional dynamic mechanical measurements.

## 4. Summary

As instrumented indentation techniques gain wider use and application, standardization efforts increase in importance. NIST personnel are involved in the ASTM Task Group E28.06.11 developing ASTM standard practices and standard test methods for instrumented indentation testing and have participated in international round robin testing. Deficiencies in current practices include the need to use reference materials for calibrations of load-frame compliance and tip shape, the lack of traceable force calibration below 10 µN, and the lack of uncertainty budget analysis related to measurement techniques and analyses. Current research at NIST is focused on independent methods for tip shape calibration, traceable calibration methods for micro-Newton and nano-Newton level forces, and applications to viscoelastic materials such as polymeric and biological materials.

## Figures and Tables

**Table 1:** Theoretical values of parameters *m* and *ε*, both of which are related to the contact geometry, for three axisymmetric tip shapes [38], where *m* is the power law exponent of Eq. (2) and *ε* is a factor used in determining the contact depth [see Eq. (6)].

Tip geometry	*m*	*ε*
Flat-ended cylindrical punch	1	1
Paraboloid of revolution	1.5	0.75
Cone	2	2 (π−2)/π

## Footnotes

1 Certain commercial equipment, instruments, or materials are identified in this paper to foster understanding. Such identification does not imply recommendation or endorsement by the National Institute of Standards and Technology, nor does it imply that the materials or equipment identified are necessarily the best available for the purpose.
